# Surgically Induced Interpregnancy Weight Loss and Prevalence of Overweight and Obesity in Offspring

**DOI:** 10.1371/journal.pone.0082247

**Published:** 2013-12-12

**Authors:** Mikaela Willmer, Daniel Berglind, Thorkild I. A. Sørensen, Erik Näslund, Per Tynelius, Finn Rasmussen

**Affiliations:** 1 Child and Adolescent Public Health Epidemiology, Department of Public Health Sciences, Karolinska Institutet, Stockholm, Sweden; 2 Institute of Preventive Medicine, Bispebjerg and Frederiksberg University Hospital, Copenhagen, The Capital Region, Denmark; 3 Division of Surgery, Department of Clinical Sciences, Danderyd Hospital, Karolinska Institutet, Stockholm, Sweden; 4 Novo Nordisk Foundation Center for Basic Metabolic Research, Faculty of Health and Medical Sciences, University of Copenhagen, Denmark; University of Cincinnati, United States of America

## Abstract

**Introduction:**

According to the fetal overnutrition hypothesis, obesity in pregnancy predisposes the offspring to obesity. Previous studies have suggested that after biliopancreatic surgery for obesity, the offspring is less likely to be obese. This study aims to further compare the BMI development of children born before and after maternal surgical weight loss.

**Method:**

Women with at least one child born before and one child born after bariatric surgery were identified by record-linkage. Information about maternal BMI was extracted from medical records, as was information about the children's BMI from birth to 10 years of age. We retrieved BMI data at four years of age for 340 children, born to 223 women (164 children born before surgery (BS), 176 children born after surgery (AS)). We evaluated prevalence of overweight/obesity and mean BMI in children born BS and AS at the ages of four, six and ten using GEE regression models. For 71 families, where we had complete data on mother and both children, we used a fixed-effects regression model to explore the association between differences in maternal BMI in w10 of the pre- and post-operative pregnancies with siblings' BMI differences at age four.

**Results:**

In no age group did we see a significantly reduced prevalence of overweight/obesity AS. For 10-year-old girls, the AS group had significantly higher rates of obesity. There was no association between differences in maternal BMI in early pregnancy and differences in siblings' BMI at four years of age (β = −0.01, CI 95% = −0.11; 0.09).

**Conclusions:**

We have been unable to demonstrate any effect of bariatric surgery on weight development in offspring. It seems unlikely that restrictive bariatric surgery conveys a protective effect in offspring with regards to obesity.

## Introduction

The prevalence of obesity (defined as a BMI≥30 kg/m^2^) has more than doubled since 1980 [1]. The prevalence of overweight and obesity among pregnant women has also increased, both in Sweden and on a global scale. Maternal obesity has numerous detrimental effects throughout conception, gestation and delivery. Compared to non-obese women, obese women have a higher risk of infertility, miscarriage, pre-eclampsia, gestational diabetes, pre- or post-term induction of labour and caesarean section [2]. Infants born to obese women are at higher risk of foetal distress, shoulder dystocia, being born large-for-gestational-age (LGA) and perinatal morbidity and mortality [2].

A woman who is obese as she enters pregnancy is at risk of hyperinsulinemia, dyslipidemia and impaired endothelial function [3]. These conditions, combined with the up-regulation of inflammatory processes which occurs concurrently with them, all put the infant at risk of childhood obesity, and of developing type 2 diabetes, cardiovascular disease and obesity later in life [4], [5]. Cnattingius *et al* have demonstrated that mothers who were themselves born LGA are at greater risk of delivering a LGA baby, compared with mothers who were born at the appropriate weight for gestational age [6]. Similarly, Ferraro *et al* showed that women who suffered from pre-pregnancy overweight or obesity, and who gained more weight than the Institute of medicine (IOM) 2009 guidelines (which recommend that an overweight woman should aim for a gestational weight gain of 15–25 lbs, or 7–11 kgs. For obese women, the recommended gestational weight gain is 11–20 lbs, or 5–9 kgs), had a strongly increased risk of delivering a LGA baby [7].

The positive association found between maternal and offspring BMI may be explained by foetal overnutrition, which occurs as the foetus is exposed to high concentrations of glucose and fatty acids in the mother's circulation. Support for this theory has been found in several studies [8], [9].

The positive association between maternal and offspring BMI may also be explained by the offspring's inheritance of the mother's obesity-related genes, or by shared familial lifestyle behaviours related to obesity. Epigenetic mechanisms have also been suggested as an explanation for this phenomenon, although the exact nature of these remain largely unknown [10].

In light of the above, it is of great importance to offer effective weight loss strategies to obese women of fertile age. However, most traditional weight loss methods – which revolve around decreased energy intake, increased physical activity and various forms of behavioural modifications – have been shown to have modest effects on long-term weight loss [11], with weight regain being the rule rather than the exception [12], [13].

Bariatric surgery has been shown to produce significant long-term weight loss, reduction in comorbidities and improvement of cardiovascular risk factors, together with low complication rates [14], [15], [16]. A number of studies have shown that women who become pregnant after undergoing bariatric surgery have improved fertility and a reduced risk of gestational diabetes, pre-eclampsia, macrosomia and hypertensive disorders, compared to obese controls. However, some studies have shown an increased risk of nutritional deficiencies and of babies being born small-for-gestational-age (SGA) [17], [18], [19].

Only a very few studies have compared BMI and prevalences of overweight and obesity in groups of children born before and after maternal weight loss from bariatric surgery, or in siblings born to the same woman before and after bariatric surgery.

Kral *et al* [20] compared rates of overweight and obesity in 217 children born to 113 women (45 born before maternal bariatric surgery, 172 born after surgery), and found that they were dramatically lower in the group of children born after surgery. A different study was later performed using the same material, but with the additional inclusion of metabolic and more detailed anthropometric data. It found significant metabolic improvements, such as greater insulin sensitivity, improved lipid profiles, lower c-reactive protein and increased ghrelin levels, in the group of children born after maternal surgery [21].

Barisione *et al* [22] asked 37 mothers, who had given birth before and after undergoing biliopancreatic diversion surgery, to retrospectively rate their children (whose current mean ages were 28.9 and 18.3 years for siblings born before and after surgery, respectively) as normal weight, overweight or obese at the ages of one, six and 12. They found that there were no differences in the mothers' ratings of the children at the ages of one and six, but that a significantly greater proportion of the children born before surgery were rated as overweight or obese at 12 years of age.

The aim of the current study was to investigate the effects of maternal interpregnancy weight loss on offspring's BMI and risk of overweight and obesity at the ages of four, six and ten. By using a cohort of Swedish women who have given birth before and after undergoing bariatric surgery we were able to analyse differences in groups of children born before and after maternal surgery, as well as perform a separate subgroup analysis in sibling pairs born to the same woman before and after bariatric surgery.

## Methods

### Ethics statement

The present study was evaluated and given ethical approval by the Stockholm Regional Ethical Review Board (ref no 2009/709-31/2).

### Sampling procedure

A database was created using record-linkage between the Swedish Medical Birth Register, which covers 99% of all births in Sweden [23], the Hospital Discharge Register, which contains information about all bariatric operations performed in Sweden, the Cause of Death Register (in order to exclude those women who had died since undergoing bariatric surgery), the Cancer Register (in order to exclude those women who had undergone gastric surgery as cancer treatment) and the Register of the Total Population, which contains data on all current residents in Sweden. This record-linkage was made possible by using the personal identification number unique to each Swedish resident.

The resulting database contained 797 women, who had all undergone bariatric surgery between 1980 and 2008, and had given birth before and after surgery. These women were sent an information letter by post, and were contacted by telephone approximately ten days later and asked whether they consented to participation. Those who accepted participation were sent written consent forms and a questionnaire to be completed and returned by post. The questionnaire contained questions on the women's and children's general health, education and their own and their children's current weight and height. Those children who were above the age of 18 were also contacted and asked to sign consent forms if they wished to participate.

**Figure 1:**
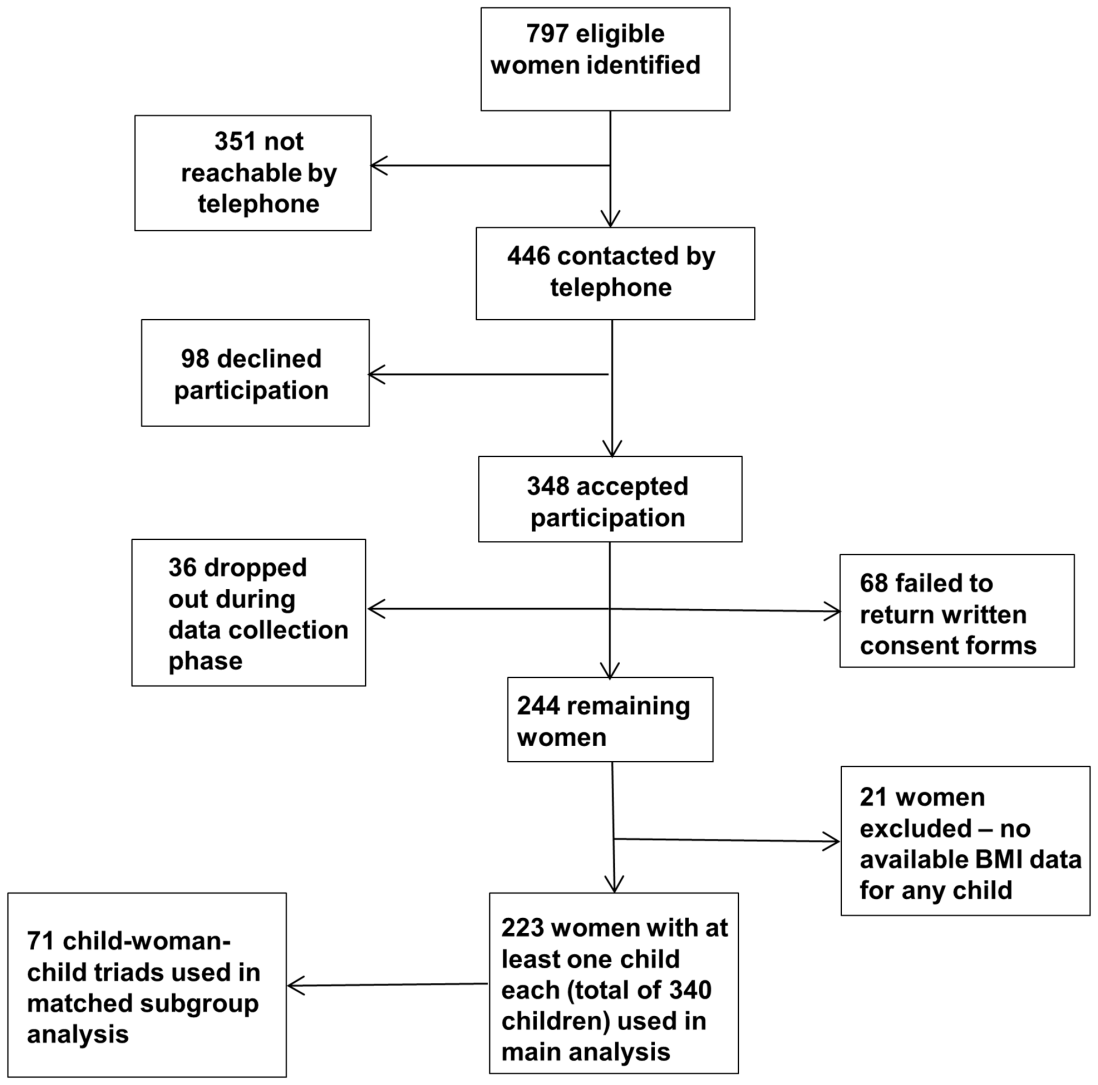
Flowchart showing the recruitment procedure and number of women who consented, declined participation, dropped out and were excluded.

We then collected data on the women's BMI at the start of their respective pregnancies, their gestational weight gain, complications during pregnancy, BMI at the time of their bariatric surgery and all available follow-up BMI data from hospital records. For the children, who were between the ages of six and 40 at the time of data collection, we retrieved growth charts from birth to ten years of age (or current age, if under 10) from child health centres, school health services and county and municipal archives. The numbers of women who declined, consented and dropped out are shown in **figure 1**. The 36 women who dropped out during the course of the data collection were deemed to have done so after failing to return their written consent forms, despite numerous reminders via telephone and post.

BMI at four years of age was chosen as the first outcome variable, since nearly all Swedish children visit their child health centre at approximately this age for a comprehensive check-up which includes measurements of height and weight. Childhood BMI has been shown to be a predictor for adolescent and adult BMI [24], [25], [26].

Since BMI was rarely measured at exactly four years of age (that is, not on the child's fourth birthday), we predicted BMI at four years using a non-parametric regression method, so called kernel smoothing [27]. For this we used the “lokern” package in the R software (R Project website. Available: http://www.r-project.org. Accessed 2013 Nov 4). A strength of this method is that it does not only rely on data on the individual level, but uses growth data for the whole population when predicting individual growth curves [27].

Thereafter, all predicted BMI values were evaluated graphically for each individual, and the values used in analyses have all been judged to have a very good fit in relation to observed BMI.

For the main analysis, we acquired BMI data at four years of age for a total of 340 children, born to 223 women. Out of these, 164 children were born before maternal surgery (BS) and 176 children were born after surgery (AS). In those families where we were able to acquire data for more than one child born before or after surgery, we restricted the analysis to the two children born closest in time BS and AS.

For 203 women, we were successful in retrieving information about the type of bariatric surgery they had undergone. The most common surgical method used was vertical banded gastroplasty (81 women, 40%), followed by gastric banding (58; 29%), Roux-en-Y gastric bypass (35; 17%), jejunoileal bypass (21; 10%) and horizontal gastroplasty (8; 4%). The surgeries were carried out between 1980 and 2006, with 1994 being the median.

The subgroup analysis of sibling pairs required complete data with regards to prepregnancy BMI and BMI data for both children. We achieved this for 71 child-woman-child triads.

### Statistical analyses

Using Cole's cutoff values for overweight and obesity [28], we calculated the prevalence of overweight and obesity at the ages of four, six and ten in the groups of children born BS and AS. We also calculated mean BMI in each group. We compared the groups with linear and logistic regression models using generalized estimating equations (GEE) to account for correlation within mothers, and with adjustments for maternal age and self-reported education, the child's birth year and birth order. We also performed the same analyses after stratifying the children by gender in each age group.

In order to explore the association between differences in maternal BMI in week 10 of the pregnancies BS and AS and differences in the siblings' BMI at four years of age, we used the 71 women for whom we had complete data, as described above. We used a fixed-effects regression model with differences in maternal BMI as the exposure variable and differences in siblings' BMI at age four as the outcome variable. This approach automatically adjusts for all potential confounders that do not vary between pregnancies. The model was further adjusted for the sex of the children, maternal age, smoking in pregnancy, birth order and maternal prepregnancy BMI. Fixed-effects models were estimated using the *xtreg* command in STATA 12.0 (Stata Corp, College Station, Texas, USA).

## Results

As expected, the mothers for which we were able to retrieve data on BMI in early pregnancy had a lower BMI at the start of their second pregnancy, as compared to their first (**table 1**). We also looked separately at those women who had undergone bariatric surgery using restrictive methods (147 women, 72%) and compared their interpregnancy weight loss with those women whose bariatric procedure had been carried out using a malabsorptive method (56 women, 28%). We found that the latter group's weight loss was larger, a mean 6.2 BMI units, as compared to 2.0 units for the restrictive surgery group. According to data from the Swedish Medical Birth Register, none of the women suffered from gestational diabetes or preeclampsia during either of their pregnancies.

**Table 1:** Characteristics of the women and their children born before and after bariatric surgery.

Variable (no of observations)	Before surgery		After surgery	
	n		n	
**Sex (340)**	164		176	
Girls (153)	87	53%	66	38%
Boys (187)	77	47%	110	63%
**Maternal age (340)**	164	24.7 (3.8)	176	33.4 (4.7)
**Smoking in pregnancy (318)**	148	57 (39%)	170	49 (29%)
**Breastfeeding (267)**	135	2.2 (1.9)	132	2.0 (1.9)
**Maternal prepregnancy BMI (271)**	130	36.0 (5.5)	141	31.7 (5.9)
BMI<25	1	1%	16	11%
BMI 25–30	11	8%	51	36%
BMI 30–35	51	39%	38	27%
BMI>35	67	52%	36	26%
**Birth weight (g) (337)**	162	3576 (600)	175	3434 (600)
Born LGA	25	16%	10	6%
Born SGA	9	6%	25	14%
**Born prematurely (<37 wks)**	14	9%	11	6%
**Macrosomia (birthweight > = 4000 g)**	44	27%	26	15%
**Time from 1^st^ pregnancy to surgery (years)**	164	4.8 (3.3)		
**Time from surgery to 2^nd^ pregnancy (years)**	176	4.03 (2.6)		

As for the children, those born BS were on average 142 grams heavier, as well as more likely to be born LGA, less likely to be born SGA, and more likely to be born to a women who had smoked during pregnancy, compared to the children born AS (**table 1**). We also looked at gender-specific prevalences of LGA and SGA births, and found that 8 (10%) of the girls and 1 (1%) of the boys were born SGA BS, as compared to 13 (20%) of the girls and 12 (10%) of the boys born AS. For LGA children, the corresponding figures were 12 (15%) of the girls and 13 (17%) of the boys BS and 6 (9%) of the girls and 4 (4%) of the boys AS.

The mean time period between the mother's surgery and her second pregnancy was four years, but there was a wide range of time periods, the shortest being 0.8 years and the longest 12.2 years.

When assessed with a linear GEE regression model adjusted for maternal age, maternal education, birth order and birth year, at no age did the children born AS have a significantly lower mean BMI than the children born BS (**table 2**).

**Table 2:** Mean BMI in groups of children born before and after maternal bariatric surgery, assessed with linear GEE regression.

	Before surgery			After surgery			GEE regression (adj^1^)		
Age group (n)	n	Mean BMI (crude)	Mean BMI (adj^1^)	n	Mean BMI (crude)	Mean BMI (adj^1^)	Diff	95% CI	p
**All 4-year-olds (340)**	164	16.9	16.8	176	17.1	17.2	0.46	−0.12; 1.03	0.117
4-year-old girls (155)	87	17.1	16.9	68	17.0	17.3	0.41	−0.76; 1.58	0.494
4-year-old boys (185)	77	16.6	16.6	110	17.2	17.2	0.60	−0.01; 1.11	0.088
**All 6-year-olds (340)**	171	17.5	17.2	169	17.8	18.1	0.87	−0.08; 1.82	0.072
6-year-old girls (157)	91	17.6	17.7	66	17.7	17.9	0.27	−1.01; 1.55	0.681
6-year-old boys (183)	80	17.0	16.7	103	17.9	18.1	1.33	−0.03; 2.68	0.055
**All 10-year-olds (272)**	157	20.6	20.3	111	21.6	21.9	1.61	0.09; 3.12	**0.038**
10-year-old girls (128)	86	20.6	20.5	42	22.1	22.4	1.90	−0.20; 4.00	0.077
10-year-old boys (144)	73	20.5	20.1	71	21.3	21.8	1.60	−0.72; 3.93	0.177

*Adjusted for maternal age, maternal self-reported education, child's birth year and birth order.* ^1^

When assessing and comparing prevalences of overweight and obesity using logistic GEE regression models, we found that no age or gender group showed a significantly lower prevalence of overweight/obesity AS (**tables 3** **and** **4**). In fact, for ten-year-old girls, the children born AS had a significantly *higher* prevalence of obesity than those born BS (**table 4**).

**Table 3:** Prevalence of overweight in groups of children born before and after maternal bariatric surgery, assessed with logistic GEE regression.

	Prevalence before surgery			Prevalence after surgery			GEE regression (adj^1^)		
Age group (n)	n	Overweight% (crude)	Overweight % (adj^1^)	n	Overweight% (crude)	Overweight % (adj^1^)	OR	95% CI	p
**All 4-year-olds (340)**	164	25.6	26.1	175	34.1	33.0	1.40	0.72; 2.71	0.323
4-year-old girls (153)	87	33.3	33.3	68	28.8	27.4	0.76	0.26; 2.21	0.610
4-year-old boys (187)	77	16.9	18.8	107	37.2	34.8	2.31	0.90; 5.97	0.083
**All 6-year-olds (340)**	171	40.9	41.9	169	44.4	43.4	1.07	0.58; 1.95	0.837
6-year-old girls (154)	91	49.8	51.7	66	43.7	41.0	0.65	0.24; 1.72	0.385
6-year-old boys (186)	80	30.0	32.2	103	45.3	43.1	1.60	0.68; 3.75	0.283
**All 10-year-olds (272)**	157	51.6	51.6	111	57.5	57.5	1.27	0.66; 2.44	0.476
10-year-old girls (128)	86	52.1	54.2	43	65.0	60.7	1.30	0.48; 3.56	0.605
10-year-old boys (139)	71	49.3	46.4	68	56.2	56.0	1.47	0.57; 3.83	0.429

1 *Adjusted for maternal age, maternal self-reported education, child's birth year and birth order.*

**Table 4:** Prevalence of obesity in groups of children born before and after maternal bariatric surgery, assessed with logistic GEE regression.

	Before surgery			After surgery			GEE regression (adj^1^)		
Age group (n)	n	Obesity % (crude)	Obesity % (adj^1^)	n	Obesity % (crude)	Obesity % (adj^1^)	OR	95% CI	p
**All 4-year-olds (340)**	164	11.0	8.7	176	11.4	13.5	1.64	0.68; 3.94	0.269
4-year-old girls (153)	87	13.6	13.8	66	11.4	12.1	0.88	0.41; 1.86	0.736
4-year-old boys (187)	77	7.8	5.8	110	10.9	12.6	2.34	0.95; 5.77	0.066
**All 6-year-olds (340)**	171	17.0	15.8	169	21.3	22.3	1.52	0.71; 3.25	0.280
6-year-old girls (154)	91	20.9	19.0	66	23.8	26.0	1.50	0.51; 4.42	0.465
6-year-old boys (186)	80	12.5	12.7	103	19.8	19.3	1.65	0.54; 5.06	0.385
**All 10-year-olds (272)**	157	17.6	16.1	111	30.1	32.8	**2.55**	**1.09; 6.00**	**0.032**
10-year-old girls (128)	86	17.4	17.1	43	38.1	38.2	**3.00**	**1.07; 8.38**	**0.036**
10-year-old boys (144)	71	17.8	14.8	68	25.4	29.2	2.38	0.58; 9.80	0.231

1 *Adjusted for maternal age, maternal self-reported education, child's birth year and birth order.*

We also looked specifically at the 71 sibling pairs where we had complete data for both children's BMI at age four and maternal prepregnancy BMI for both pregnancies. Mean prepregnancy BMI for the BS and AS pregnancies were 36.7 and 31.0, respectively. The children's BMI at four years of age were 16.8 BS and 17.0 AS (corresponding z-scores were −0.1 and 0.0).

When assessing the 71 child-women-child triads with complete data, there were no significant associations between differences in maternal BMI in week 10 of the pregnancies BS and AS and differences in the siblings' BMI at four years of age (**table 5**). Further adjustment for prepregnancy BMI and for time elapsed between surgery and the second pregnancy did not change the results significantly. We also tried the same model with the children's BMI converted into z-scores (standard deviation scores). We did this both with internal standardisation and with standardisation against a British reference population, as described by Vidmar *et al* [29]. This did not result in any change in the results (data not shown).

**Table 5:** Results of fixed-effects regression model exploring the association between differences in maternal BMI in week 10 of pregnancy and differences in siblings' BMI at four years of age.

Diff BMI 4 years	Model 1			Model 2			Model 3			Model 4			Model 5		
n = 142	β	95% CI	P	β	95% CI	p	β	95% CI	p	β	95% CI	p	β	95% CI	p
Diff BMI wk 10 of preg	−0.01	−0.08; 0.06	0.77	−0.01	−0.08; 0.06	0.79	0.01	−0.08; 0.11	0.78	−0.01	−0.10; 0.09	0.91	−0.01	−0.11; 0.09	0.84
Sex of siblings				0.08	−0.70; 0.85	0.84	0.05	−0.73; 0.84	0.89	0.05	−0.73; 0.82	0.90	0.02	−0.81; 0.85	0.96
Birth order							0.26	−0.51; 1.03	0.50	−0.88	−2.44; 0.69	0.27	−1.02	−2.65; 0.61	0.21
Mother's age										0.12	−0.02; 0.26	0.10	0.14	−0.01; 0.29	0.07
Smoking in pregnancy													0.51	−0.80; 1.82	0.44

In addition to the above, we also tried stratifying the analyses by surgery type (malabsorptive and restrictive). This made no significant changes to any of the results shown in tables 2–5 (data not shown).

## Discussion

This study has been unable to demonstrate that bariatric surgery has a positive effect on the obesity prevalence of female patients' future offspring. The results show no reductions in prevalence of overweight or obesity, nor in BMI, between the children born BS and AS. This is somewhat surprising, as the only other comparable study shows such a major difference between the two groups [20]. In the study by Kral *et al*, 60% of the 45 children born before maternal surgery were overweight or obese, compared to 35% of the 172 children born after maternal surgery. The corresponding figures for the four-year-olds in our study are 37% overweight or obese before surgery, compared to 46% after surgery.

There are some differences in the design and analysis approach between the present study and that by Kral *et al*, which may help to explain the differences in results. It did not compare the children's BMI at any specific age, but made use of z-scores to compare the BMI of children from ages 2–18. The study also partly relied on self-reported data. Only 34 of the 113 participating women had given birth both before and after bariatric surgery.

Our cohort includes many women who have undergone mainly restrictive surgery (VBG and GB), many of whom did not lose a large amount of weight between pregnancies. The women who participated in the study by Kral *et al* had a greater starting BMI and a greater mean weight loss (from BMI 48 to 31) than the women in our study (from 36 to 32). However, our regression model (**table 5**) examines the association between the magnitude of difference in maternal BMI and that of siblings' BMI at age four, so even if there was only an association in cases of large differences in maternal BMI, the model should have been able to detect it. The confidence interval for the fully adjusted model (**table 5**) is very narrow (−0.11; 0.09), further strengthening our results.

There is also a possibility that it is the metabolic effects of bariatric surgery, rather than the actual weight loss, that are of importance in terms of effects on the children's BMI. Roux-en-Y gastric bypass and biliopancreatic diversion have been shown to affect insulin regulation to a greater extent than restrictive surgery [30], [31]. In the article by Kral *et al*, all patients had undergone biliopancreatic diversion.

When comparing our results to those found by Barisione *et al*, one should bear in mind that that the data collection method used (asking mothers to retrospectively rate their children as normal weight, overweight or obese) may be somewhat unreliable, as mothers have been shown to be unable to rate even their child's *current* weight correctly [32], [33]. Nonetheless, the authors did not find any differences in the mothers' ratings of the children's weight status at one and six years of age, which correlates with the findings in the present study. However, they did find that a greater proportion of the children born BS were rated as overweight or obese at 12 years of age, compared to the children born AS. They also measured height and weight of the adult children who were between 21 and 25 years of age at the time of the data collection (36 children born BS and 18 born AS), and found that those born AS had a significantly lower BMI. This implies that a possible preventive effect of maternal bariatric surgery may not become evident until later in life. However, as pointed out in an editorial comment to the article, the sample born AS contained a much greater proportion of women, and as the authors did not correct for this, there is a possibility that it may have distorted the results [34].

If we return to the results of the present study, we see that for ten-year-old girls, the children born AS had significantly higher rates of obesity than those born BS (17% BS vs 38% AS). It is possible that this finding is associated to the lower birth weight and higher proportion of SGA children born AS. It has been shown that being born SGA, and experiencing rapid weight gain postnatally (catch-up growth), is associated with obesity in later life [35], [36]. There were proportionally more girls born SGA after maternal surgery in our sample (20% as compared to 10% of the boys), which may explain why we only saw increased obesity prevalence in girls.

Overall, the children in our sample suffered from levels of overweight and obesity which were much higher than those found in the Swedish general population, where rates of childhood overweight and obesity have been found to be approximately 16–22% for overweight and 3–5% for obesity [37], [38], [39]. This in itself is not surprising, since the children in the current study all have an inherited predisposition for obesity (from at least one parent) and are very likely to have been exposed to unfavourable environmental factors during childhood (such as unhealthy eating habits and lack of physical activity).

The present study has several strengths and weaknesses. It relies exclusively on data extracted from hospital records and records kept by child health centres and school health services, and is therefore likely to have a high degree of accuracy. Furthermore, all BMI comparisons used for the children are based on measurements taken at the same age for all children – i.e. four-year-olds are only compared to other four-year-olds. Also, the design used for the analysis of the 71 women with complete data automatically adjusts for all potential confounders that do not vary between pregnancies.

When it comes to potential weaknesses, the relatively small sample size should be mentioned. One of the main reasons for this was that we were unable to reach almost half of the original sample by telephone or regular mail. Although we cannot be entirely certain that this has not been a source of selection bias – for example, those who cannot be reached by telephone may not own one because of low socioeconomic status – but we feel that it is unlikely to have unduly affect the results, as we also tried contacting the women by regular mail.

Naturally, it would have been desirable to retrieve complete data for all child-mother-child triads in the cohort, so that the sibling pair analysis could have contained more observations. However, it proved very difficult to obtain older, archived child health centre records. After exploring all avenues during a data collection period of more than two years, we were only able to achieve this for 71 women and the children they gave birth to before and after surgery. This prompted us to focus on group comparisons of children born before and after maternal bariatric surgery, in addition to the analysis of sibling pairs born to the same woman.

We did not have access to reliable data on paternity, which is a potential problem as the sibling pairs in the subgroup analysis (**table 5**) may have different fathers. However, this would have been a more serious problem if our results had indicated that the children born BS were heavier than those born AS. If so, one might have argued that the mother may have had an obese partner at the time of her first pregnancy (as BMI tends to be similar in couples [40], [41]), only to switch to a less obese partner as she lost weight after her bariatric surgery. The lower BMI in the children born AS could then be explained by the differing paternity. As our results show no such tendencies, however, this lack of data is perhaps of less concern.

Finally, the material is very heterogeneous in terms of surgical method used, and many of the participants have undergone restrictive surgery such as vertical banded gastroplasty and gastric banding. These women may have experienced less weight loss and may also not have had the same advantageous effects on their insulin regulation as patients treated with Roux-en-Y gastric bypass or biliopancreatic diversion.

In conclusion, the present study found no decreased rates of overweight or obesity in children born after maternal restrictive bariatric surgery compared to children who were born prior to surgery. It is possible that only methods which cause direct changes in metabolic regulation have an effect on the overweight/obesity rates of the patients' future children. However, a significant interpregnancy weight loss was achieved in our subjects without any effect on weight development in the offspring, which casts some doubt on the hypothesis that bariatric surgery conveys a protective effect on overweight or obesity in the children of operated women.
